# Optimal method for ablation of atypical AVNRT

**DOI:** 10.1186/s12872-023-03305-9

**Published:** 2023-05-19

**Authors:** Amir Aslani, Shahab Shahrzad, Mehdi Bazrafshan, Mahdi Rahmanian, Reza Fakhar, Kasra Pirahesh, Hanieh bazrafshan, Hamed Bazrafshan

**Affiliations:** 1 Cardiology Department, Shiraz University of Medical Sciences, Shiraz, Iran; 2 Fars Heart Foundation, Kowsar Hospital, Shiraz, Iran; 3 Cardiovascular Research Center, Shiraz University of Medical Sciences, Shiraz, Iran; 4 Tehran University of Medical Sciences, Tehran, Iran; 5 Neurology Research Center, Shiraz University of Medical Sciences, Shiraz, Iran

**Keywords:** Atypical AVNRT, Ablation, Slow pathway

## Abstract

**Background:**

Considering that ablation of atypical AVNRT may be unsuccessful after ablation at the right posterior septum, in this study, we aimed to present an optimal method for ablation of atypical AVNRT. Also, we evaluated the efficacy of this technique for preventing recurrences.

**Methods:**

This is a prospective, double-center study. It was conducted on 62 patients with atypical AVNRT referred for radiofrequency ablation. The patients were randomly divided into two groups before ablation: 1-Group A (n = 30): treated with conventional ablation at the anatomic area of the slow pathway; 2-Group B (n = 32): ablation was done 2 mm higher in the septum during fluoroscopy.

**Results:**

The mean age of patients in groups A and B were 54 ± 11.7 and 55 ± 12.2, respectively (P = 0.43). In group A, ablation was successful in 24 (80%) patients following right-sided slow pathway ablation, and the remaining patients required further treatment with either a left-side approach (N = 4, 13.3%) or ablation of additional regions (N = 2, 6.7%). In group B, ablation was successful in all patients. After a 48-month follow-up, recurrence of symptomatic atypical AVNRT was detected in 4 (13.3%) patients of group A and none of group B patients (p < 0.001).

**Conclusion:**

In patients with atypical AVNRT, ablation 2 mm above the conventional area is more promising regarding success rate and recurrence of the arrhythmia.

## Introduction

AV nodal reentrant tachycardia (AVNRT), the most common type of supraventricular tachycardia, is characterized by tachycardia with a narrow QRS of supraventricular origin, sudden termination, and a heart rate of 150–250 beats/min [1]. AVNRT denotes reentry in the area of the AV node and is categorized as typical or atypical. Although several models have been proposed to explain the mechanism of the arrhythmia in the context of the complex anatomy and the anisotropic properties of the AV node and its atrial extensions, the actual circuit of AVNRT remains elusive. In typical AVNRT (also described as slow-fast AVNRT), the impulse travels over the slow pathway toward the ventricles and returns via the fast pathway to the atria. In atypical ANVRT (also called fast-slow AVNRT), the impulse travels via the fast pathway toward the ventricles and returns via the slow pathway to the atria. In an electrophysiological study, AVNRT is divided into typical (slow-fast) and atypical (fast-slow) according to the relative duration of atrio-His (AH) versus His-atrial (HA) conduction and earliest atrial activation site during the tachycardia [1–3]. Slow pathway ablation with a RF catheter is considered the fundamental treatment approach of AVNRT.

Most AVNRT cases can be treated with slow pathway ablation from the right part of the inferoseptal segment [2, 3]. Rare cases of either typical or atypical AVNRT are refractory to standard right-sided ablation [4]. Thus, they require left-sided ablation via the left atrium or coronary sinus [5–12]. RF ablation has a success rate of 95–98% in achieving long-term cure in typical AVNRT. Literature data on atypical AVNRT, in contrast with its typical form, are insufficient because of its low prevalence. Atypical AVNRT has been identified as a predictor of lower ablation success rates, and the optimal method of catheter ablation is not established. In most case reports, ablation of atypical AVNRT was performed by other techniques. Otomo et al. have reported that slow pathway ablation within the coronary sinus is effective for treating atypical AVNRT after an unsuccessful right-sided ablation [13]. In another report, a case of atypical AVNRT with multiple fast-slow pathways was successfully ablated via a left-sided approach [14]. In most published reports, ablation of atypical AVNRT was guided by indicating the slow pathway with an evaluation of retrograde atrial activation [13, 15–21].

Regarding ablation of atypical AVNRT may be unsuccessful after ablation at the right posterior septum, we hypothesized that patients with atypical AVNRT might have a structural and/or electrophysiological remodeling of the AV nodal tissue, resulting in a superior displacement of slow pathway. Therefore, in this prospective study, we aimed to present an optimal method for ablating atypical AVNRT. Also, we evaluated the efficacy of this technique for preventing recurrences.

## Materials and methods

### Study population

This research is a prospective, double-center study. It was conducted on 62 patients with atypical AVNRT referred for radiofrequency ablation (2013–2019). All patients with symptomatic AVNRT with at least one episode every two months were included in this study. Those patients who underwent ablation therapy and developed recurrence were excluded. Demographic and clinical data were recorded before ablation. The patients were randomly divided into two groups before ablation based on random digit number: 1-Group A (n = 30): treated with “conventional ablation” at the anatomic area of the slow pathway; 2-Group B (n = 32): ablation was done 2 mm higher in the septum (during fluoroscopy). This study was approved by the Ethics Committee of the Shiraz University of Medical Sciences (IR.SUMS.MED.REC.1396.04), and written informed consent was obtained from the subjects.

### Definition of atypical AVNRT

Atrio-ventricular node reentrant tachycardia (AVNRT) was diagnosed by fulfillment of standard criteria during an electrophysiologic study with atrial/ventricular pacing maneuvers: 1-Typical (slow-fast) AVNRT was characterized by AH/HA ratio > 1, and HA ≤ 70 ms, 2-Atypical AVNRT was defined by delayed retrograde atrial activation with HA > 70 ms. If the AH was < 200 ms and the AH < HA, the atypical AVNRT was diagnosed as fast-slow. If AH > 200 ms and AH > HA, the atypical AVNRT was diagnosed as slow-slow.

### Electrophysiologic study

All of the antiarrhythmic drugs were stopped five lifetimes before the study. Five electro catheters were inserted into the heart, three via the left femoral vein and two via the right femoral vein. Three quadripolar catheters from the left femoral vein were placed in RA-RV-HIS location, and one decapolar catheter from the right femoral vein was placed into the CS. An ablation catheter was inserted into the right atrium to map and ablate the slow pathway.

### Slow pathway ablation

The patients were randomly divided into two groups before ablation: 1-Group A (n = 30): treated with “conventional ablation” at the anatomic area of the slow pathway; 2-Group B (n = 32): ablation was done 2 mm higher in the septum (during fluoroscopy):

In group-A, conventional anatomic slow pathway RF ablation was performed according to established techniques. In brief, a conventional 4-mm ablation catheter (Boston Scientific Stinger Ablation Catheter, D Curve 7 F) was positioned at the postero-septal (infero-septal) part of the tricuspid annulus until an A-V ratio of < 1 was recorded. The ablation catheter was kept at the ostium of the coronary sinus (CS) as visualized in the left anterior oblique view (mapping was not performed at the mid or anterior septum). When multi-component signals or low-amplitude potentials were obtained, RF current, 30 to 40 W aimed at a temperature of 55–60 °C, was delivered for up to 30 s until a junctional rhythm with 1:1 retrograde V-A conduction was observed. RF delivery was immediately stopped if V-A conduction was not seen. Once junctional rhythm with V-A conduction was recorded, energy delivery was continued for 30 s. Following RF ablation, arrhythmia induction with the use of Isuprel was performed. Successful ablation was defined as RF-induced junctional rhythm (conducted to the atria) and non-inducibility of AVNRT with programmed stimulation during Isuprel infusion.

In group B, RF ablation was performed at 2 mm higher in the septum with the same techniques and catheters used for patients in Group-A.

### Statistical analysis

Continuous data were expressed as Mean ± SD. The normality of data was analyzed using the Kolmogorov-Smirnoff test. Independent sample t-test and chi-square tests were used for bivariate analysis. P value 0 < 0.05 was considered to be statistically significant. The statistical software IBM SPSS Statistics for Windows version 22.0 (IBM Corp. Released 2013, Armonk, New York) was used for the statistical analysis.

## Results

### Baseline characteristics

A total of 62 patients with atypical AVNRT were studied. Group A (n = 30, 15 male, age 54 ± 11.7 years) were treated with “conventional ablation” at the anatomic area of the slow pathway. Group B (n = 32, 13 male, age 55 ± 12.2 years) were treated with ablation 2 mm higher in the septum. There were no significant differences in age and sex between the two groups (P = 0.51 and P = 0.43, respectively).

### Electrophysiological findings

In group A, 26 of the 30 patients (86.6%) had fast-slow AVNRT, and 4 patients (13.3%) had slow-slow AVNRT. In group B, 27 of the 32 patients (84.3%) had fast-slow AVNRT and 5 patients (15.6%) had slow-slow AVNRT. Electrophysiological findings during arrhythmia are shown in Table 1.

**Table 1:** Electrophysiological and ablation findings in patients with atypical AVNRT

Item	Group A (n = 30)	Group B (n = 32)	*P*
AH (ms)	124.4 ± 58.6	128.9 ± 63.8	0.76
HA [His] (ms)	210.8 ± 64.5	222.5 ± 72.3	0.87
Fluoroscopy Time (min)	22.1 ± 3.4	24.5 ± 3.6	0.88
Radiofrequency Time (min)	6.1 ± 4.6	5.8 ± 4.5	0.77
Burn Time (min)	4.9 ± 2.2	4.3 ± 2.6	0.75
Junctional Rhythm (During Radiofrequency)	30 patients	32 patients	0.99
V-A Block (During Radiofrequency)	4 patients	6 patients	0.99
AV Block	0	0	0.99

### Ablation characteristics

Group-A patients were treated with “conventional ablation” at the anatomic area of the slow pathway as described. This was successful in 24 patients (80%). In 4 patients, a left-sided approach was accomplished following unsuccessful right-sided slow pathway ablation. In 2 patients, additional lesions were needed in the roof of the proximal coronary sinus following unsuccessful ablation.

Group-B patients were treated with ablation 2 mm higher in the septal area during fluoroscopy. This was successful in all patients without the need for additional lesions at other sites (p = 0.01 for the percentages of successful right-sided slow pathway ablation). The ablation characteristics of the two groups are highlighted in Table 1.

### Patients follow-up

48-month follow-up was completed in groups A and B patients. Recurrence of symptomatic atypical AVNRT was detected in 4 (13.3%) patients of group A and none of the group B patients (p < 0.001 for the percentages of recurrences in group-A versus group B). No case of AV block was noted during follow-up in either group-A or group B patients (Table 2).

**Table 2:** Follow-up results of patients

Item	Group A (n = 30)	Group B (n = 32)	p
Recurrence	4 (13.3%)	0 (0%)	< 0.001
AV-block	0 (0%)	0 (0%)	0.99

## Discussion

In contrast with its typical form, Literature data on atypical AVNRT need to be revised because of its low prevalence. Atypical AVNRT has been identified as a predictor of lower ablation success rates, and the optimal method of catheter ablation still needs to be established. The same anatomic site and nature of the pathways involved in the atypical AVNRT have yet to be recognized, and efforts to provide a reasonable hypothesis based on anatomic or anisotropic models have been made [22]. Retrograde atrial activation in the atypical AVNRT begins well after ventricular activation, indicating that retrograde conduction is slower than antegrade conduction. The earliest retrograde atrial activation is typically reported at the base of the Koch triangle, near the CS ostium [23]. Detailed mapping of retrograde atrial activation in large series of patients, however, has produced variable results, with eccentric atrial activation at the lower septum or even the distal coronary sinus [11]. Thus, the success rate of ablation of atypical AVNRT is variable according to the mapped location of the retrograde slow pathway. Regarding ablation of atypical AVNRT may be unsuccessful after ablation at the right posterior septum, we hypothesized that patients with atypical AVNRT might have a structural and/or electrophysiological remodeling of the AV nodal tissue, resulting in a superior displacement of slow pathway. This hypothesis was formed according to our prior clinical observations. Regarding those observations, in atypical AVNRT cases, multiple RF delivery was required in the conventional area. Also, we found that burning 2 mm above the conventional area led to less RF delivery, an increased success rate of stopping the arrhythmia, and decreased arrhythmia recurrence. In this prospective study, we treated atypical AVNRT cases with ablation 2 mm higher in the septal area (2 mm above the conventional anatomic area of the slow pathway). This approach was successful in all patients without additional lesions at other sites. None of the patients experienced recurrence during the 48-month follow-up. Also, no case of AV block was noted during the follow-up. These interesting results were significantly superior to our control patients, who were treated with conventional ablation at the anatomic area of the slow pathway.

In contrast to our result, Katritsis et al. studied 2079 patients with AVNRT, of whom 113 were cases of atypical AVNRT or coexistent typical and atypical AVNRT. They found that ablation in the conventional area is the treatment of choice in typical and atypical patients [24]. The contrast between their result and ours can be associated with selecting atypical patients. Our cases with atypical AVNRT did not have a coexistent typical arrhythmia. Typical coexistent AVNRT may have a confounding effect that requires further studies.

Our study had some notable strengths, especially 48-month patient follow-up. The limitation of our study was the small sample size. We recommend further studies with more cases of atypical AVNRT.

## Conclusion

In patients with atypical AVNRT, ablation 2 mm above the conventional area seems to be more promising regarding success rate and recurrence of the arrhythmia.

## Data Availability

SPSS data of the participants can be requested from the authors. Please write to the corresponding author if you are interested in such data.
